# Isolation of Low-Abundant Bacteroidales in the Human Intestine and the Analysis of Their Differential Utilization Based on Plant-Derived Polysaccharides

**DOI:** 10.3389/fmicb.2018.01319

**Published:** 2018-06-19

**Authors:** Huizi Tan, Jianxin Zhao, Hao Zhang, Qixiao Zhai, Wei Chen

**Affiliations:** ^1^State Key Laboratory of Food Science and Technology, Jiangnan University, Wuxi, China; ^2^School of Food Science and Technology, Jiangnan University, Wuxi, China; ^3^National Engineering Research Center for Functional Food, Jiangnan University, Wuxi, China; ^4^International Joint Research Laboratory for Probiotics, Jiangnan University, Wuxi, China; ^5^Beijing Innovation Center of Food Nutrition and Human Health, Beijing Technology and Business University, Beijing, China

**Keywords:** xylan, isolation, Bacteroidales, transcriptomics, metabolomics

## Abstract

Bacteroidales are the most abundant Gram-negative bacteria flourished in the human intestine with great underlying benefits to be discovered and developed as the next-generation probiotics. However, the traditional isolation method limits the mining of low-abundant species. In this study, modified selective medium was established using xylan as the sole carbohydrate source to enrich low-abundant species such as *Prevotella copri* and *Bacteroides xylanisolvens* from healthy human fecal samples. The growth rate, transcriptomics, and metabolomics profiles of the enriched low-abundant species were then evaluated. The considerable upregulated genes encoding xylan-associated hydrolysis and transportation, along with the increased xylose production detected in the culture of the enriched Bacteroidales strains based on xylan, were considered as positive proof of the feasibility of the modified methodology.

## Introduction

Species or strains with health beneficial functions in addition to the traditional probiotics such as Lactobacillus and Bifidobacterium are under exploiting to be novel new-generation probiotics, of which Bacteroidales species, the most abundant Gram-negative bacteria flourished in human intestine, are one of the most potential candidates (O’Toole et al., 2017). For instance, the capsular polysaccharides produced by *Bacteroides fragilis* ATCC25285 have been found to prevent intestinal inflammatory disease induced by pathogenic *Helicobacter hepaticus* (Mazmanian et al., 2008), and the tumor-specific Thomsen-Friedenreich antigen (TFα) secreted by *B. ovatus* D-6 with capsular polysaccharides is capable of sufficiently activating specific anti-TFα antibodies *in vivo* and thereby potentially prevent cancer development (Ulsemer et al., 2013).

The discovery of beneficial characteristics depends on an adequate number of isolated strains. The well acknowledged techniques for isolating Bacteroidales resort to the Brucella laked blood, kanamycin, and vancomycin medium (LKV) (Zitomersky et al., 2011), through which the difficulties of purifying certain species are determined by their abundance in the sample and thereby the Bacteroidales species in the healthy human intestine with relatively high abundance of over 1%, which corresponds to *B. uniformis*, *B. vulgatus*, *B. caccae*, *Parabacteroides distasonis*, *P. merdae*, and *Alistipes putredinis* as identified by the next-generation sequencing techniques (Tap et al., 2009; Qin et al., 2010; Jeffery et al., 2016), are much easier to be obtained. However, low-abundant species also exhibit beneficial talents, such as *B. xylanisolvens*, which was confirmed to promote the suppression of cancer development via inducing the TFα-specific immunoglobulin M serum antibodies (Ulsemer et al., 2016), and *B. acidifaciens* JCM10556, which facilitates the maintenance of intestinal mucosa by stimulating the secretion of immunoglobulin A and thereby elevating pathogens that have breached the epithelial walls (Yanagibashi et al., 2013). In addition, fewer strains of low-abundant Bacteroidales and their genome sequences have been available for further differential functional analysis. Therefore, methodologies for purifying these low-abundant Bacteroidales species are worth exploration.

Bacteroidales possess unique and powerful carbohydrates-utilization systems and establish syntrophic interaction networks (Rakoff-Nahoum et al., 2014, 2016). Multiple polysaccharide utilization loci (PULs) composed of large numbers of glycoside hydrolases (GHs) built around orthologs of the starch utilization system (Sus), were identified in *B. thetaiotaomicron*, which is considered as the type species for investigating the polysaccharide degradation abilities within the Bacteroidales family (Bolam and Sonnenburg, 2011). And various utilization strategies can be selectively activated based on the carbohydrates available in the environment (Sonnenburg et al., 2005). In this study, the differential responses to carbohydrates, including xylan, rhamnose, and mannitol, which are preferred by only a few relatively low-abundant Bacteroidales species according to Bergey’s Manual, were considered as key points for optimizing the enrichment effects based on the classic isolation method. Differential transcribed genes and the intracellular metabolites of the isolated Bacteroidales species were monitored by the techniques of RNA sequencing and gas chromatography so as to describe the underlying utilization pathways and thereby assisted the verification of the reliability of the isolation method.

## Materials and Methods

### Medium

Media used in this study include isolation agar plate, which was composed of carbon-free (cf) medium-peptone 20 g/L, yeast extracts 5 g/L, NaCl 5 g/L, K_2_HPO_4_ 0.05 g/L, KH_2_PO_4_ 0.05 g/L, L-cysteine HCl 1 g/L, hemin 0.01 g/L, vitamin K1 2 mg/L, kanamycin 0.1 g/L, and vancomycin 0.75 mg/L, and supplemented with 5% selected carbohydrate (xylan, rhamnose, or mannitol), 0.012 g/L bromocresol purple, and 15–20g/L agar; isolation control agar plate (CTRL), composed of brain heart infusion supplemented with 0.01 g/L hemin, 2 mg/L vitamin K1, 0.1 g/L kanamycin, 0.75 mg/L vancomycin, and 15–20g/L agar; and BHIS medium, composed of brain heart infusion supplemented with 0.01 g/L hemin and 2 mg/L vitamin K1.

Peptone and yeast extracts were purchased from Oxoid, United Kingdom; kanamycin, vancomycin, hemin, vitamin K1, rhamnose, and mannitol were purchased from Sangon Biotech, China; xylan was purchased from Sigma-Aldrich, United States; brain heart infusion was a product from Hopebio, China; and the rest reagents were obtained from Shanghai Hushi Laboratorial Equipment Co., Ltd., China.

### Isolation and Identification of Intestinal Bacteroidales Strains

Seven fecal samples were collected from healthy volunteers (three from healthy children and four from healthy elderly donors) in Shandong province, China. Samples were serially diluted with phosphate-buffered saline solution (pH 7.2) supplemented with 1 g/L L-cysteine HCl and spread on cf-xylan, cf-rhamnose, cf-mannitol, and CTRL agar plates. After 48-h anaerobic cultivation at 37°C, 10–12 single colonies with yellow pigments and different morphologies from cf-xylan, cf-rhamnose agar or cf-mannitol medium, which located on the plates containing 30–300 colonies, were purified by streaking on new agar plates, respectively. Another 10–12 single colonies with different morphologies from CTRL medium were also purified, followed by the subculture in the BHIS medium. Strains were identified by 16s rRNA sequencing (BGI Shenzhen, China) and blasting against the NCBI database^1^.

The efficiency of the method for isolating low-abundant Bacteroidales species from human fecal samples described above was verified on three bacteria consortium samples prepared with the isolated Bacteroidales strains under various diversities (**Table 1**).

**Table 1:** The composition of three Bacteroidales consortium (BCS) for verifying the isolation methodology.

Consortium	BCS1		BCS2		BCS3	
Strain info	unit/10^8^cfu	%	unit/10^8^cfu	%	unit/10^8^cfu	%
*Bacteroides uniformis* HCM-XY15	4	15.38	4	15.38	4	14.81
*Bacteroides vulgatus* HCM-XY3	3	11.54	0	0.00	0	0.00
*Bacteroides thetaiotaomicron* HCK-B2	4	15.38	4	15.38	4	14.81
*Parabacteroides distasonis* HCM-4h5MIC2	4	15.38	4	15.38	4	14.81
*Bacteroides caccae* ELH-2.5MIC3	0	0.00	0	0.00	3	11.11
*Parabacteroides merdae* ELI-BHI4	0	0.00	3	11.54	0	0.00
*Bacteroides ovatus* HCM-XY1	2	7.69	1	3.85	2	7.41
*Bacteroides eggerthii* HCK-xy6	2	7.69	1	3.85	1	3.70
*Bacteroides xylanisolvens* HCM-XY17	1	3.85	1	3.85	1	3.70
*Prevotella copri* ELH-xy3	1	3.85	2	7.69	2	7.41
*Bacteroides fragilis* HCK-B3	2	7.69	1	3.85	2	7.41
*Bacteroides cellulosilyticus* ELH-2.5MIC4	1	3.85	2	7.69	2	7.41
*Bacteroides dorei* HCK-B6	1	3.85	2	7.69	0	0.00
*Odoribacter splanchnicus* ELH-2.5MIC5	1	3.85	0	0.00	1	3.70
*Bacteroides salyersiae* ELI-BHI9	0	0.00	1	3.85	1	3.70
Total	26	100	26	100	27	100

### Growth Curve Analysis

*Prevotella copri* ELH-XY3, *B. xylanisolvens* HCM-XY17, and *B. uniformis* HCM-XY15, which were isolated as described above, were subcultured using carbon-free broth medium supplemented by 5% xylan or xylose with 2% overnight culture, respectively. Absorbance at 600 nm was measured every 60 min. The experiments were carried out in triplicate. The growth curve was generated by GraphPad Prism software (GraphPad Software Inc., United States).

### Draft Genome Sequencing

The genomic DNA of *P. copri* ELH-XY3, *B. xylanisolvens* HCM-XY17, and *B. uniformis* HCM-XY15 was extracted by Novogene Bioinformatics Technology Co. Ltd. (Beijing, China) from cell pellets grown in BHIS broth at the stationary phase, and fragmented using a Covaris ultrasonic into pieces with 350 bp or 6 kb for library preparation and Illumina HiSeq sequencing. Sequencing data were assembled by SOAPdenovo (version 2.04) (Li et al., 2008, 2010), followed by modification using krskgf (version 1.2) and gapclose (version 1.12). Gene functions were predicted by GeneMarkS (version 4.17)^2^ (Besemer et al., 2001). The draft genome sequences were used as reference genomes for transcriptomic analysis.

### Transcriptomic Analysis

Cells of *P. copri* ELH-XY3, *B. xylanisolvens* HCM-XY17, and *B. uniformis* HCM-XY15 grown in cf-xylan were harvested at OD600 of 0.83, 0.89, and 0.69, respectively, and cells of the three strains grown in cf-xylose were collected at OD600 of 0.75, 0.75, and 0.64, respectively, for transcriptomic profiling. Three biological replicates were used to evaluate the reproducibility of the RNA-Seq analysis. The pellets were collected for total RNA extraction by Novogene Bioinformatics Technology Co. Ltd. (Beijing, China). rRNA was removed using Ribo-zero kit. The library was constructed by mRNA fragments, cDNA reverse transcription, PCR amplification, and purification with AMPure XP beads. The library was sequenced by Illumina HiSeq sequencing. Clean data were obtained by filtering adaptor sequences, followed by location analysis using Bowtie2 (Langmead et al., 2009) to ensure that the mapping rate to the reference genome was over 70%. HTSeq (Anders et al., 2015) was used to analyze the expression levels of genes with FPKM (expected number of fragments per kilobase of transcript sequence per millions base pairs sequenced) values over 1. The correlation in expression of genes between biological replicates was examined by calculating the Pearson correlation coefficient using R script^3^. DESeq (Anders and Huber, 2010) was used to determine the differentially expressed genes, of which the log_2_ (fold change) of the transcription in bacterial cells grown in xylan relative to that in xylose was over 1 and the *p*-value was less than 0.05, after normalization with FPKM.

### Analysis of Metabolites

The metabolites produced by *P. copri* ELH-XY3, *B. xylanisolvens* HCM-XY17, and *B. uniformis* HCM-XY15 during fermentation in xylan or xylose were investigated by gas chromatography time-of-flight mass spectrometry (GC-TOF MS) as described (Shin et al., 2010a), with modifications. 10 ml bacterial culture of *P. copri* ELH-XY3, *B. xylanisolvens* HCM-XY17, and *B. uniformis* HCM-XY15 grown in xylan at OD600 of 1.66, 1.78, and 1.38, respectively, along with the cells fermented in xylose at OD600 of 1.5, 1.5, and 1.28, respectively, were quenched individually with 30 ml of 20% methanol (Merck, Germany) prepared in 0.9% sodium chloride solution at -4°C for 10 min before centrifuging at 10,000 ×*g* under the same temperature condition for 10 min. The cell pellets were washed twice with 5 ml of 4% methanol prepared in 0.9% sodium chloride solution and centrifuged again at 10,000 ×*g* for 10 min. The total metabolites were extracted from the cell pellets by resuspending with 3 ml acetonitrile-methanol-aqua solution (2:2:1, v/v/v) and three cycles of freezing in liquid nitrogen for 3 min and thawing at -20°C for 20 min, before centrifuged at 10,000 × *g* and -20°C for 10 min. The extraction step was repeated with 1 ml of acetonitrile-methanol-aqua solution (2:2:1, v/v/v, Merck, Germany), and the supernatant was combined for vacuum-drying after being supplemented with 50 μl of 0.1032 mg/ml heneicosanoic acid (SCRC, Shanghai, China) as retention index marker. The residuals were reserved for detecting the total protein content, as instructed by the BCA protein assay kit (Tiangen Biotech, China). The metabolite extracts were reconstituted with 100 μL of 10 mg/ml methoxyamine hydrochloride (TCI chemicals, Japan) in pyridine, and incubated at 37°C for 90 min, followed by derivatization with 40 μl *N*-methyl-*N*-(trimethylsilyl) trifluoroacetamide with 1% trimethylchlorosilane (Sigma-Aldrich, United States) at 37°C for 30 min. The metabolites mixture samples were ready for applying to the gas chromatography–mass spectrometer (TSQ^TM^ 8000 Evo, Thermo Scientific) with the RTX-5MS column (30 m × 0.25 mm, 0.25 μm of film thickness, Restek, United States) before being centrifuged at 10,000 ×*g* for 10 min. The working temperature started at 70°C and ramped to 230°C at a rate of 5°C per minute, followed by further increasing to 320°C at a rate of 90°C per minute, and finally held for 6 min. Mass spectra were acquired by establishing a scan range of 85–500 m/z, electron impact at 70 eV for ionization, and the temperature of ion source at 300°C. The experiments were carried out in triplicates. Ultra-pure water from Milli-Q system (Millipore Corp., United States) was used for preparing all the aqua solutions mentioned throughout the experiment of metabolomics analysis.

The detected metabolite candidates were identified according to the NIST mass spectral database library with similarity index of over 800. The integral area was generated as the production yield, followed by normalization of the internal standard and total protein content of each sample, and log transformation afterward. The validated data was visualized as heatmaps with hierarchical clustering analysis and principal co-ordinates analysis (PCoA) using R script (see footnote 3).

## Results

### Xylan Exhibited an Outstanding Enrichment Effect on Relatively Low-Abundant Intestinal Bacteroidales Species

From the results of the bacterial isolation from both fecal samples and the Bacteroidales consortium, as shown in **Tables 2A,B**, more probable number of colonies corresponding to *B. ovatus*, *B. uniformis*, and *B. thetaiotaomicron* could be picked out from the control medium, which was a modified version of the standard selective medium for general Bacteroidales species. More *B. ovatus* and *P. distasonis* were purified based on the cf-mannitol medium, while more *B. ovatus* and *B. thetaiotaomicron* were isolated from the cf-rhamnose medium compared with the standard method, without significant differences observed. Xylan exhibited the best enrichment effect among the three carbohydrates for low-abundant Bacteroidales species, from which *P. copri* and *B. xylanisolvens* were much easier to be obtained. It is also more likely to obtain *B. uniformis* colonies, one of the major Bacteroidales species in the human intestine, from the cf-xylan medium. In order to further investigate the differential carbohydrate utilization abilities of these xylanolytic bacteria, the profiles of growth rate, transcriptomics and metabolomics were analyzed based on xylan and xylose fermentation.

**Table 2:** Species isolated from **(A)** fecal samples and **(B)** Bacteroidales consortium using medium with xylan (cf-xylan), rhamnose (cf-rhamnose) and mannitol (cf-mannitol) as the sole carbohydrates, and the BHIS control medium (CTRL).

Species	cf-xylan	cf-rhamnose	cf-mannitol	CTRL
**(A)**				

*B. ovatus*	48.03 ± 27.79	66.11 ± 22.89	60.74 ± 29.72	36.90 ± 19.10
*B. thetaiotaomicron*	3.78 ± 6.66	23.54 ± 23.59	10.61 ± 20.76	15.28 ± 14.24
*B. fragilis*	u.d.	u.d.	u.d.	13.57 ± 14.27
*B. uniformis*	17.22 ± 15.03	u.d.	8.56 ± 9.30	10.88 ± 15.05
*B. eggerthii*	3.78 ± 6.66	1.19 ± 2.95	u.d.	5.93 ± 7.19
*P. distasonis*	7.36 ± 6.99	2.25 ± 3.62	11.43 ± 17.26	5.45 ± 8.64
*B. vulgatus*	3.57 ± 8.75	3.57 ± 8.75	u.d.	3.23 ± 5.35
*P. merdae*	u.d.	u.d.	u.d.	2.60 ± 6.36
*B. cellulosilyticus*	u.d.	3.34 ± 5.46	4.64 ± 7.41	2.60 ± 6.36
*B. salyersiae*	u.d.	u.d.	1.43 ± 3.50	1.30 ± 3.18
*P. copri*	13.89 ± 28.24	u.d.	2.60 ± 6.36	1.30 ± 3.18
*B. dorei*	u.d.	u.d.	u.d.	0.95 ± 2.33
*B. xylanisolvens*	2.38 ± 5.83	u.d.	u.d.	u.d.

**(B)**				

*B. uniformis*	27.27 ± 7.42	u.d.	6.06 ± 8.57	21.21 ± 11.34
*P. distasonis*	15.15 ± 11.34	9.09 ± 7.42	45.45 ± 12.86	18.18 ± 19.64
*B. fragilis*	u.d.	u.d.	u.d.	15.15 ± 4.29
*B. cellulosilyticus*	18.18 ± 7.42	18.18 ± 12.86	24.24 ± 8.57	15.15 ± 11.34
*B. thetaiotaomicron*	6.06 ± 8.57	54.54 ± 19.64	6.06 ± 8.57	9.09 ± 7.42
*B. ovatus*	12.12 ± 11.34	15.15 ± 21.43	15.15 ± 11.34	9.09 ± 7.42
*B. salyersiae*	u.d.	u.d.	u.d.	6.06 ± 4.29
*B. eggerthii*	u.d.	u.d.	u.d.	3.03 ± 4.29
*B. dorei*	u.d.	u.d.	u.d.	3.03 ± 4.29
*B. caccae*	u.d.	u.d.	3.03 ± 4.29	u.d.
*B. vulgatus*	u.d.	3.03 ± 4.29	u.d.	u.d.
*B. xylanisolvens*	6.06 ± 4.29	u.d.	u.d.	u.d.
*P. copri*	15.15 ± 11.34	u.d.	u.d.	u.d.

*Data are presented in percentage of the number of colonies corresponding to certain species based on the total number of colonies picked from individual samples placed on each medium. All data are given as mean ± SD, no significant differences were observed between groups. u.d. is short for undetected.*

### Comparison of Bacterial Growth Based on Xylan and Xylose

**FIGURE 1:**
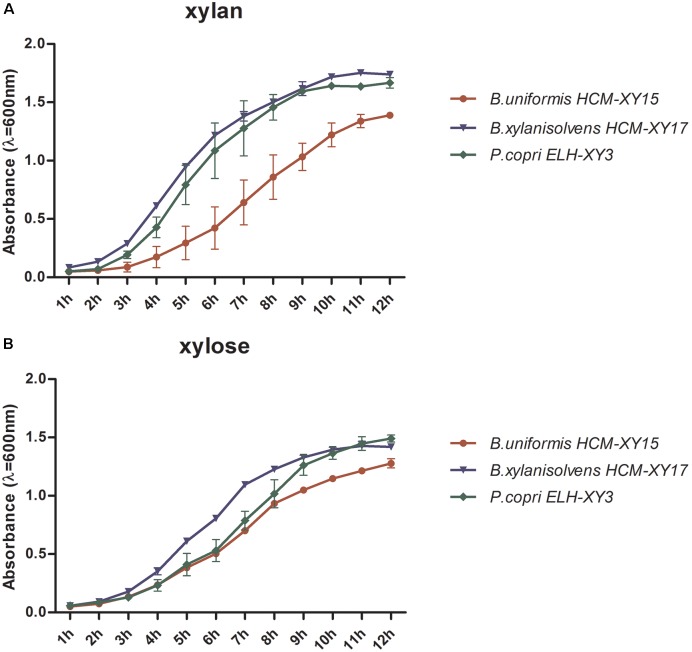
Comparison of the growth rate of *P. copri* ELH-XY3, *B. xylanisolvens* HCM-XY17, and *B. uniformis* HCM-XY15 in medium with sole carbohydrate of **(A)** xylan or **(B)** xylose. All data are given as mean ± SD.

The culture of *P. copri* ELH-XY3, *B. xylanisolvens* HCM-XY17, and *B. uniformis* HCM-XY15 could reach early stationary phase after 10-h fermentation in either cf-xylan or cf-xylose broth medium, respectively. *P. copri* ELH-XY3 and *B. xylanisolvens* HCM-XY17 exhibited better growth patterns compared to *B. uniformis* HCM-XY15 in xylan (**Figure 1A**), while the growth rates became close among the three strains in xylose (**Figure 1B**). Based on the growth profiles of the cells in both medium, culture in the mid-logarithmic phase was chosen for transcriptomic analysis, and late-logarithmic phase was chosen for metabolomics analysis to allow the accumulation of metabolites.

### Comparison of Transcriptome Profiles During Fermentation Based on Xylan and Xylose

The sequencing results showed high correlation in the expression of genes in the biological replicates fermented in both xylan and xylose. An average of 19,816,191 and 16,911,855 of cDNA reads were obtained from *P. copri* ELH-XY3 cells fermented in xylan and xylose, respectively, with mapping rate of 99.17% and 99.08%; cDNA reads for *B. xylanisolvens* HCM-XY17 were 16,810,671 and 17,525,322 with mapping rates of 99.42% and 99.45%; and for *B. uniformis* HCM-XY15 the averages reads were 15,058,070 and 17,174,780 with mapping rates of 99.36% and 98.64%. The largest amount of differentially activated genes was found in *P. copri* ELH-XY3, with 832 genes differentially expressed in total during growth with xylan relative to xylose. In *B. xylanisolvens* HCM-XY17 and *B. uniformis* HCM-XY15, 505 and 337 of differentially transcribed genes were revealed, respectively.

Three hundred and fifty-three genes of *P. copri* ELH-XY3 were induced more than fivefold in comparison of the responses to xylan over xylose (Supplementary Table S1), most of which were annotated as hypothetical protein based on the NCBI database. It is notable that a series of carbohydrates utilization enzymes like Sus particularly SusC and SusD, which are the essential signatures of PULs, GH, glycosyl transferase, carbohydrate esterase, and transporters, were also highly enriched. Six genes with predicted functions related to xylan hydrolysis, including xylan esterase, endo-xylanase, and arabinosidase (GH43), were revealed to be significantly upregulated. However, only 11 genes of *P. copri* ELH-XY3 were repressed over fivefold, and none of the xylan utilization-related genes were involved (Supplementary Table S2). These results indicated that *Prevotella copri* would set up a transcriptional program during xylan fermentation, which convokes considerable carbohydrate utilization genes, particularly those associated with xylan.

For *B. xylanisolvens* HCM-XY17, 64 genes were transcribed more than fivefold during growth in xylan relative to xylose (Supplementary Table S3), and seven were repressed greater than fivefold (Supplementary Table S4). Most of the upregulated genes were identified to be Sus, GH, or hypothetical protein.

Compared with the two Bacteroidales strains described above, *B. uniformis* HCM-XY15 displayed less significant differentiation in the upregulated transcription but more in the downregulation while utilizing xylan compared to xylose, among which 52 genes were induced more than fivefold (Supplementary Table S5) and 22 were repressed more than fivefold (Supplementary Table S6). Conversely, however, BuniforGM001890, with the predicted function of xylose isomerase and BuniforGM000205 encoding arabinofuranosidase, was repressed by about fivefold during cultivation in xylan.

### Comparison of Metabolic Products Throughout Fermentation Based on Xylan and Xylose

**FIGURE 2:**
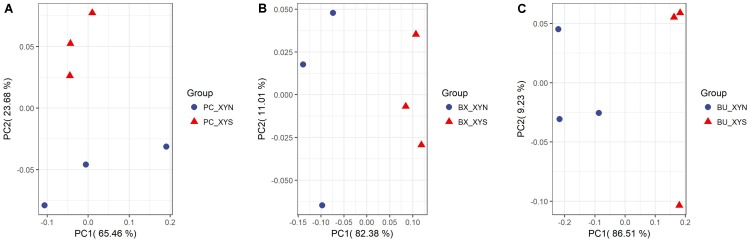
PCoA plots by analyzing metabolites produced by **(A)** *P. copri* ELH-XY3 (Pc), **(B)** *B. xylanisolvens* HCM-XY17 (Bx), and **(C)** *B. uniformis* HCM-XY15 (Bu) from medium with sole carbohydrate of xylan (xyn) or xylose (xys) in three biological replicates, respectively.

**FIGURE 3:**
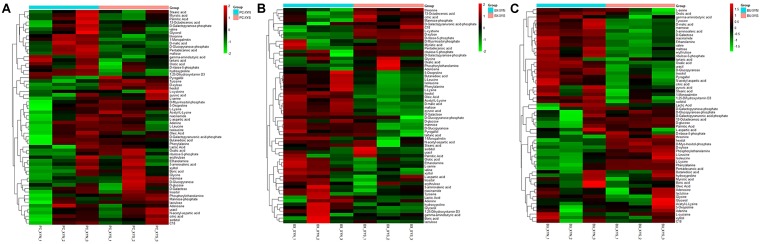
Hierarchical clustering analysis of detected global metabolites produced by **(A)** *P. copri* ELH-XY3 (Pc), **(B)** *B. xylanisolvens* HCM-XY17 (Bx), and **(C)** *B. uniformis* HCM-XY15 (Bu) from medium with sole carbohydrate of xylan (xyn) or xylose (xys) in three biological replicates, respectively.

A total of 63 metabolites involved in major pathways such as the TCA cycle, purine and pyrimidine metabolism, along with amino acids, organic acid, and sugars were detected from the culture *P. copri* ELH-XY3, *B. xylanisolvens* HCM-XY17, and *B. uniformis* HCM-XY15 in both xylan and xylose. The products profiles were clearly distinguished from the same Bacteroidales strain grown in the different carbohydrates, or from different strains in xylan, according to the PCoA shown in **Figure 2**. There are nine metabolites shared between the three Bacteroidales strains, including stearic acid, malic acid, gamma-aminobutyric acid (GABA), tartaric acid, orotic acid, dihydroxyvitamin D3, valine, 1-monopalmitin, and maltose, which performed higher levels of secretion in xylan over xylose (**Figure 3**). Glucopyranose was also enriched either in the form of sugar or sugar-phosphate.

**FIGURE 4:**
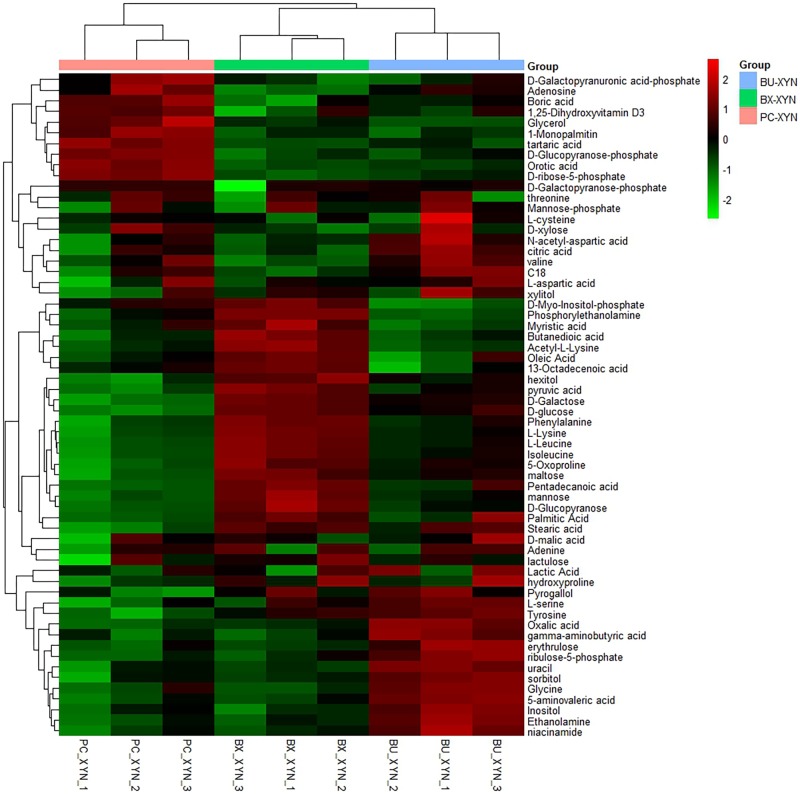
Hierarchical clustering analysis of detected global metabolites during xylan (xyn) fermentation by *P. copri* ELH-XY3 (Pc), *B. xylanisolvens* HCM-XY17 (Bx) and *B. uniformis* HCM-XY15 (Bu) in three biological replicates, respectively.

Three major clusters were generated by comparing the differential metabolites from the three Bacteroidales strains cultured in xylan using hierarchical clustering analysis (**Figure 4**). The first cluster was composed of organic acids such as tartaric acid and orotic acid, fatty acids such as 1-monopalmitin, amines such as adenosine, and especially sugars such as xylose, which exhibited more expression in *P. copri* than in *B. xylanisolvens* and *B. uniformis*. While there were more of the metabolites from *B. xylanisolvens* and *B. uniformis* corresponding to categories of amino acids, fatty acids, or organic acids, and monosaccharides other than xylose, according to the other two clusters in the heat map.

## Discussion

**FIGURE 5:**
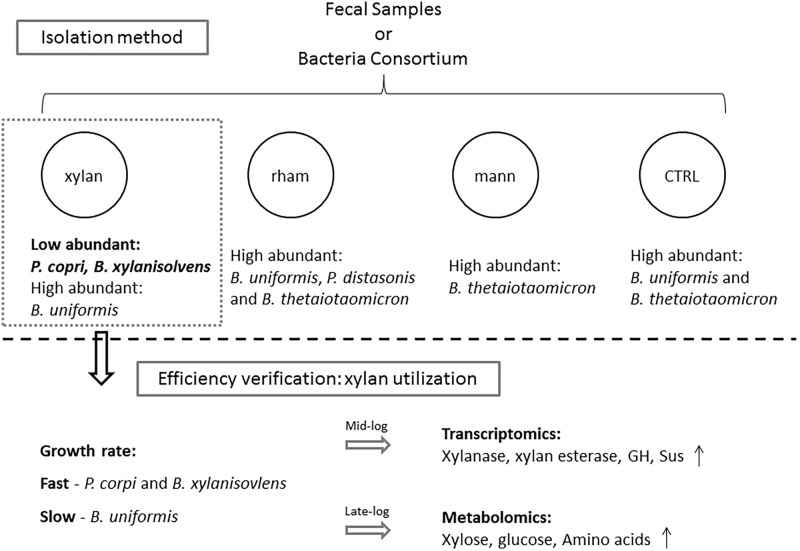
The framework of this study. Medium with xylan, mannitol (mann) or rhamnose (rham) as the sole carbohydrate were used for isolating low abundant Bacteroidales species from fecal samples and bacteria consortium, traditional Bacteroidales isolation medium was used as control (CTRL), among which medium containing xylan exhibited the best enrichment effects on low abundant candidates, such as *P. copri* and *B. xylanisolvens*, the efficiency of which was confirmed by evaluating the differential utilization responses to xylan by the enriched species via examining the growth rate in xylan and the detection of profiles of transcribed genes and metabolites during the fermentation.

In the present study, we conducted a pilot investigation of establishing an optimized isolation method for low-abundant Bacteroidales species from fecal samples using plant-derived polysaccharide as the sole carbohydrate. The isolation efficiency was verified by evaluating the growth rate, transcriptomics, and metabolomics profiles of the enriched low-abundant species (**Figure 5**).

The selective medium comprised kanamycin and vancomycin as Bacteroidales selective antibiotics, hemin and vitamin K1 as Bacteroidales growth factors, bromocresol purple as pH indicator, which could turn the agar plate from purple (pH 7.0) to yellow (around pH 5) with the acid rapidly produced by the bacterial colonies during carbohydrate utilization, K_2_HPO_4_ and KH_2_PO_4_ as the balancing salts and peptone and yeast extracts as nitrogen sources. Xylan, out of the three potential carbohydrates according to Bergey’s Manual, exhibited the best effects in enriching low-abundant Bacteroidales species, particularly *P. copri* and *B. xylanisolvens*, from both fecal samples and artificial Bacteroidales cocktails. *B. uniformis*, one of the major Bacteroidales species in the human intestine, was also concentrated, but the hysteretic growth rate based on xylan may indicate that the *P. copri* and *B. xylanisolvens* involved in this study are actually better xylan utilizers.

Carbohydrate utilization associated genes transcription are the essential indicators for investigating the capabilities of polysaccharides degradation. The mid-log phase was chosen for transcription analysis, as a latest research suggested that more genes at this time-point would be involved in breaking down polysaccharides than at the late-log phase (Despres et al., 2016). The positive effect of xylan on isolating low-abundant Bacteroidales from biological samples was, to some extent, verified by the transcriptomics profiles, as the substantial amount of the upregulated genes for more than fivefold with predicted functions of GH, esterase and transferase were identified in *P. copri* ELH-XY3 and *B. xylanisolvens* HCM-XY17 relative to the cultivation in xylose. In particular, the PcopriGM000867 encoding endo-1, 4-beta-xylanase, and PcopriGM000851, 0852, 860, 0867, and 2978 encoding acetyl xylan esterase, as the crucial enzymes during depolymerization of xylan (Gilbert, 2010; Girio et al., 2010), were prominently upregulated in *P. copri*. Moreover, the significantly repressed xylan hydrolysis-associated genes revealed in *B. uniformis* HCM-XY15 were in accordance with the impaired growth conditions in the cf-xylan medium and may demonstrate the less priority of xylan as energetic source for *B. uniformis* compared to *P. copri* and *B. xylanisolvens*.

Up till now, several models for degrading xylan by various Bacteroides species have been discovered. For instance, the PULs 73 with indispensable GH30 and GH98 of *B. ovatus* ATC8483 takes the responsibility on breaking down glucuronoarabinoxylans (Rogowski et al., 2015). A combination of PUL43 who binds to xylan and PUL70 who completes the degradation and facilitates the transportation in *B. xylanisolvens* XB1A orchestrates the oat-spelt xylan (OSX) utilization (Despres et al., 2016). The sequencing depth in this study limited the cluster analysis; however, the transcriptomics outcome suggested that *P. copri* ELH-XY3 performed a PUL43-like strategy composed of β-1,4-xylanase (PcopriGM000867) with GH3 (PcopriGM000863), GH43 (PcopriGM000861), GH31 (PcopriGM000862) and the SusCD-pairs around, which were markedly induced during the cultivation with xylan as the sole carbohydrate source. Furthermore, one of the open reading frames encoding GH family 5 in *P. bryantii* B14 synergistically promoted the release of xylose from wheat arabinoxylan (WAX) with β-D-xylosidase and α-L-arabinofuranosidase. Although resulted in different products (Dodd et al., 2010), the collaboration scheme was confirmed in *B. eggerthii* DSM20697 and *B. intestinalis* DSM17393, which may also demonstrate the dominance in growth of *P. copri* ELH-XY3 based on xylan, as the genes PcopriGM000002 and PcopriGM000839 encoding GH5 were significantly upregulated. It is unexpectedly to define few xylan utilization-related genes to be upregulated in the typical xylanolytic *B. xylanisolvens*, which could be on account of improper type of xylan used, just as J. Despres once reported that the upregulated genes of *B. xylanisolvens* XB1A grown in OSX turned to be repressed in WAX (Despres et al., 2016); or may be due to the mismatches during blasting from the draft genome.

Moreover, metabolites are important factors for investigating degradation capabilities. Xylan can be broken down to xylose and a variety of oligosaccharides by beta-1, 4-xylanase from bacteria. In our study, *P. copri* ELH-XY3 achieved the most xylose production among the three Bacteroidales strains, which was in concert with the transcriptomics results and the outstanding growth rate. *B. xylanisolvens* HCM-XY17 and *B. uniformis* HCM-XY15 achieved more production of amino acid and simple saccharides such as glucose and galactose. The high levels of glucose and/or xylose indicated that the pentose phosphate pathway was adopted during xylan utilization by Bacteroidales, as these monosaccharides are considered as major control points (Shin et al., 2010b). The relatively high yield of dihydroxyvitamin D3 and orotic acid which was also termed as vitamin B13 detected by GC-TOF MS revealed the capabilities of sterol hydroxylation by *P. copri* ELH-XY3. The superior secretion of ribose-phosphate might be related with nucleotides synthesis through a *de novo* pathway (Dawes and Mizrahi, 2001; Rolfes, 2006).

While investigating the impact of carbohydrates on metabolites profiles, most of the common increased metabolites shared within the three strains grown in xylan corresponded to fatty acid category. Shin et al. (2010b) suggested that this may be due to the accumulation of degradative enzymes attached to the surface, which are the essential steps for breaking down complex polysaccharides. The higher yields of GABA produced by Bacteroidales in xylan may also worth further investigating as GABA plays a dominant role in inhibiting transmission in the central nervous system (Tan and O’Toole, 2015); Furthermore, it is likely to be the ingredient xylose in the recipe which caused the relatively higher levels in bacteria grown in xylose medium.

Although Bacteroidales in general demonstrates outstanding carbohydrate utilization capabilities, xylan degradation is not the universal talent (Zhang et al., 2014), which gives the chance of the xylanolytic characteristics of *P. copri* and *B. xylanisolvens* to help bacterial reproduction and enrichment in circumstances with xylan as the sole carbohydrate source and implement the selective isolation from complex communities. Furthermore, *P. copri* was proved to be associated with autoimmunity regulation in human, such as rheumatoid arthritis (Hofer, 2014); One of the *B. xylanisolvens* strains was recently authorized as starters for fermentation of pasteurized milk products by the European Commission for its beneficial characteristics (Brodmann et al., 2017); Also, xylanolytic characteristics could be considered to be health-promoting as the simple glycan released from xylan could help the growth of probiotics such as Bifidobacterium (Rogowski et al., 2015). Thus, the established isolation method in this study for the low-abundant xylanolytic intestinal commensals could promote the further investigations in host-microbes interactions. However, the isolation efficiency of this method may be challenged by the diversity of the samples, which keeps altering due to the various living habitats of the hosts.

## Author Contributions

HT carried out the experiment and drafted the manuscript. JZ and HZ provided the essential reagents and materials, and participated in the bacterial isolation assay, growth rate assay, metabolomics assays, and analyzed the data. QZ conceived of the study and managed the project design. QZ and WC helped to revise the manuscript. All authors read and approved the final manuscript.

## Conflict of Interest Statement

The authors declare that the research was conducted in the absence of any commercial or financial relationships that could be construed as a potential conflict of interest.

## Footnotes

1 https://blast.ncbi.nlm.nih.gov/Blast.cgi

2 http://topaz.gatech.edu/GeneMark/

3 http://www.r-project.org
