# Supplementary material for: Isolation of Low-Abundant Bacteroidales in the Human Intestine and the Analysis of Their Differential Utilization Based on Plant-Derived Polysaccharides
Source: Front Microbiol. 2018 Jun 19;9:1319. doi: 10.3389/fmicb.2018.01319 (PMC6018473; doi:10.3389/fmicb.2018.01319)
Supplement: Supplementary file 1 [file Table_1.DOCX]

Table S1 Genes induced over 5-fold in *P. copri* ELH-XY3 during fermentation in xylan relative to xylose. Genes are listed by magnitude of induction. Gene annotation was carried out by blastp against the NCBI database.

| Gene_id | Fold Change (log2) | p-value | annotation |
| --- | --- | --- | --- |
| PcopriGM002127 | 7.6671 | 1.47E-26 | hypothetical protein |
| PcopriGM002818 | 7.597 | 1.19E-27 | outer membrane protein (SusC) |
| PcopriGM002128 | 7.3808 | 1.23E-22 | hypothetical protein |
| PcopriGM002821 | 7.2411 | 1.22E-22 | membrane-anchored phosphodiesterase |
| PcopriGM000258 | 7.1042 | 5.91E-21 | hypothetical protein |
| PcopriGM002103 | 7.1042 | 5.91E-21 | hypothetical protein |
| PcopriGM000233 | 7.0467 | 1.05E-205 | TonB-dependent receptor |
| PcopriGM002847 | 7.0005 | 2.76E-18 | hypothetical protein |
| PcopriGM000849 | 6.9381 | 1.18E-17 | protein/carbohydrate esterase-related protein |
| PcopriGM000986 | 6.8894 | 3.55E-17 | type I phosphodiesterase/nucleotide pyrophosphatase |
| PcopriGM003309 | 6.8632 | 2.79E-18 | hypothetical protein |
| PcopriGM000480 | 6.7958 | 2.72E-16 | carbohydrate-active enzyme |
| PcopriGM003169 | 6.792 | 1.48E-17 | hypothetical protein |
| PcopriGM000384 | 6.6082 | 1.18E-14 | hypothetical protein |
| PcopriGM003175 | 6.5981 | 1.43E-14 | ADP-ribosylglycohydrolase |
| PcopriGM000338 | 6.583 | 1.38E-15 | hypothetical protein |
| PcopriGM000360 | 6.5642 | 2.93E-29 | beta-galactosidase |
| PcopriGM000337 | 6.5594 | 3.55E-29 | alpha-L-rhamnosidase |
| PcopriGM002514 | 6.5468 | 3.74E-14 | hypothetical protein |
| PcopriGM002130 | 6.5363 | 4.54E-14 | hypothetical protein |
| PcopriGM002124 | 6.5257 | 5.50E-14 | MFS transporter |
| PcopriGM000523 | 6.4761 | 1.15E-14 | ligand-gated channel protein |
| PcopriGM000266 | 6.3824 | 6.68E-14 | hypothetical protein |
| PcopriGM002288 | 6.3571 | 1.03E-12 | hypothetical protein |
| PcopriGM002117 | 6.3451 | 1.26E-12 | glycosyl transferase |
| PcopriGM002123 | 6.3386 | 1.47E-13 | hypothetical protein |
| PcopriGM003290 | 6.2837 | 3.40E-12 | beta-lactamase |
| PcopriGM003294 | 6.247 | 7.23E-13 | sensor histidine kinase |
| PcopriGM002405 | 6.2456 | 6.20E-12 | hypothetical protein |
| PcopriGM001993 | 6.2351 | 5.08E-24 | hypothetical protein |
| PcopriGM003127 | 6.2111 | 1.32E-12 | site-specific integrase |
| PcopriGM002810 | 6.1797 | 1.70E-11 | hypothetical protein |
| PcopriGM003172 | 6.1797 | 1.70E-11 | hypothetical protein |
| PcopriGM001057 | 6.1742 | 2.42E-12 | hypothetical protein |
| PcopriGM001679 | 6.1742 | 2.42E-12 | ammonium transporter |
| PcopriGM000571 | 6.0778 | 7.65E-22 | primase |
| PcopriGM000995 | 6.0711 | 1.23E-11 | primase |
| PcopriGM002980 | 6.0711 | 8.18E-32 | Trehalase |
| PcopriGM002108 | 6.0676 | 8.66E-11 | hypothetical protein |
| PcopriGM003168 | 6.0529 | 1.06E-10 | hypothetical protein |
| PcopriGM003005 | 6.0442 | 1.85E-11 | Beta-galactosidase |
| PcopriGM000232 | 6.0383 | 2.13E-70 | outer membrane protein for nutrient uptake (SusD) |
| PcopriGM003351 | 6.0305 | 2.28E-11 | site-specific integrase |
| PcopriGM002112 | 6.0167 | 4.77E-21 | hypothetical protein |
| PcopriGM000857 | 5.9928 | 2.43E-10 | hypothetical protein |
| PcopriGM002289 | 5.9887 | 4.23E-11 | hypothetical protein |
| PcopriGM003295 | 5.9887 | 4.23E-11 | hypothetical protein |
| PcopriGM000261 | 5.9774 | 2.99E-10 | hypothetical protein |
| PcopriGM003234 | 5.946 | 4.53E-10 | hypothetical protein |
| PcopriGM002817 | 5.9456 | 7.88E-11 | hypothetical protein |
| PcopriGM002118 | 5.9301 | 5.58E-10 | glycosyl transferase, family 1 |
| PcopriGM002848 | 5.9301 | 5.58E-10 | hypothetical protein |
| PcopriGM003254 | 5.914 | 6.88E-10 | conjugal transfer protein TraG |
| PcopriGM003063 | 5.9062 | 1.03E-28 | carboxypeptidase regulatory protein |
| PcopriGM000569 | 5.8977 | 8.48E-10 | hypothetical protein |
| PcopriGM002107 | 5.8645 | 1.29E-09 | hypothetical protein |
| PcopriGM002141 | 5.8476 | 1.59E-09 | hypothetical protein |
| PcopriGM000002 | 5.8306 | 1.97E-09 | glycoside hydrolase, family 5 |
| PcopriGM002113 | 5.8241 | 4.21E-10 | hypothetical protein |
| PcopriGM003291 | 5.8133 | 2.43E-09 | hypothetical protein |
| PcopriGM002094 | 5.8081 | 5.20E-10 | hypothetical protein |
| PcopriGM000491 | 5.7781 | 3.71E-09 | site-specific integrase |
| PcopriGM002106 | 5.7592 | 9.82E-10 | hypothetical protein |
| PcopriGM003346 | 5.7236 | 7.04E-09 | tetracycline-inactivating monooxygenase Tet(X) |
| PcopriGM002116 | 5.705 | 8.73E-09 | hypothetical protein |
| PcopriGM003003 | 5.705 | 8.73E-09 | Beta-galactosidase |
| PcopriGM002786 | 5.6913 | 2.31E-09 | hypothetical protein |
| PcopriGM000259 | 5.6671 | 1.34E-08 | hypothetical protein |
| PcopriGM000952 | 5.6561 | 6.84E-17 | rhamnogalacturonan lyase |
| PcopriGM003173 | 5.6477 | 1.66E-08 | hypothetical protein |
| PcopriGM003311 | 5.6281 | 2.07E-08 | DNA-binding protein |
| PcopriGM002114 | 5.6017 | 6.77E-09 | glycosyl transferase, family 1 |
| PcopriGM002110 | 5.5675 | 3.96E-08 | glycosyl transferase, family 2 |
| PcopriGM002994 | 5.5642 | 1.05E-08 | hypothetical protein |
| PcopriGM000264 | 5.5468 | 4.93E-08 | hypothetical protein |
| PcopriGM003213 | 5.5257 | 6.13E-08 | hypothetical protein |
| PcopriGM000214 | 5.5061 | 2.01E-08 | hypothetical protein |
| PcopriGM002823 | 5.5043 | 7.63E-08 | hypothetical protein |
| PcopriGM000993 | 5.4826 | 9.51E-08 | hypothetical protein |
| PcopriGM002131 | 5.4761 | 4.02E-15 | ATP-dependent helicase |
| PcopriGM003207 | 5.466 | 3.12E-08 | toxin-antitoxin system Bro |
| PcopriGM000996 | 5.3824 | 7.54E-08 | Bacteriophage protein |
| PcopriGM002807 | 5.3607 | 2.35E-20 | hypothetical protein |
| PcopriGM002512 | 5.3451 | 3.60E-07 | hypothetical protein |
| PcopriGM002845 | 5.3451 | 3.60E-07 | hypothetical protein |
| PcopriGM002132 | 5.3219 | 6.10E-26 | hypothetical protein |
| PcopriGM000422 | 5.3209 | 4.50E-07 | L-arabinosidase, GH43 |
| PcopriGM002109 | 5.3209 | 4.50E-07 | glycosyl transferase, family 11 |
| PcopriGM003296 | 5.3209 | 4.50E-07 | nuclease |
| PcopriGM003279 | 5.2935 | 1.84E-07 | relaxase |
| PcopriGM000854 | 5.2232 | 2.52E-24 | outer membrane protein (SusC) |
| PcopriGM003165 | 5.2196 | 1.11E-06 | ATP-binding protein |
| PcopriGM001396 | 5.1931 | 1.39E-06 | hypothetical protein |
| PcopriGM002096 | 5.1931 | 1.39E-06 | hypothetical protein |
| PcopriGM000625 | 5.1662 | 1.74E-06 | hypothetical protein |
| PcopriGM001157 | 5.1107 | 2.75E-06 | hypothetical protein |
| PcopriGM000265 | 5.1042 | 1.68E-22 | restriction endonuclease |
| PcopriGM003070 | 5.0976 | 1.13E-06 | hypothetical protein |
| PcopriGM000479 | 5.0844 | 7.24E-12 | hypothetical protein |
| PcopriGM002979 | 5.0622 | 8.29E-17 | alpha-L-rhamnosidase |
| PcopriGM002115 | 5.0577 | 1.14E-11 | polysaccharide pyruvyl transferase |
| PcopriGM003209 | 5.0442 | 1.29E-36 | hypothetical protein |
| PcopriGM000949 | 5.0232 | 5.49E-06 | hypothetical protein |
| PcopriGM002515 | 5.0167 | 2.54E-16 | hypothetical protein |
| PcopriGM003188 | 4.9792 | 6.25E-16 | ATPase AAA |
| PcopriGM002392 | 4.9618 | 8.73E-06 | hypothetical protein |
| PcopriGM000478 | 4.9601 | 9.81E-16 | outer membrane protein for nutrient uptake (SusD) |
| PcopriGM000901 | 4.9301 | 1.10E-05 | hypothetical protein |
| PcopriGM002120 | 4.9301 | 1.10E-05 | hypothetical protein |
| PcopriGM002653 | 4.9301 | 1.10E-05 | helicase |
| PcopriGM002139 | 4.8977 | 1.39E-05 | hypothetical protein |
| PcopriGM000947 | 4.8708 | 2.18E-10 | hypothetical protein |
| PcopriGM002129 | 4.8645 | 1.76E-05 | hypothetical protein |
| PcopriGM003126 | 4.8645 | 1.76E-05 | hypothetical protein |
| PcopriGM000861 | 4.8585 | 1.64E-23 | glycoside hydrolase, family 43 |
| PcopriGM001054 | 4.8554 | 4.34E-19 | hypothetical protein |
| PcopriGM000306 | 4.8398 | 9.05E-06 | hypothetical protein |
| PcopriGM000434 | 4.832 | 8.57E-19 | ATP-binding protein |
| PcopriGM002757 | 4.8306 | 2.23E-05 | hypothetical protein |
| PcopriGM002819 | 4.8293 | 1.87E-14 | Starch-binding associating with outer membrane |
| PcopriGM000267 | 4.8241 | 3.06E-27 | restriction-modification system |
| PcopriGM000860 | 4.7958 | 2.82E-05 | acetyl xylan esterase |
| PcopriGM001144 | 4.7958 | 2.82E-05 | hypothetical protein |
| PcopriGM002142 | 4.7958 | 2.82E-05 | hypothetical protein |
| PcopriGM000231 | 4.7702 | 8.28E-51 | toxin-antitoxin system Bro |
| PcopriGM002091 | 4.7602 | 3.57E-05 | hypothetical protein |
| PcopriGM002752 | 4.7602 | 3.57E-05 | hypothetical protein |
| PcopriGM000257 | 4.7236 | 4.53E-05 | hypothetical protein |
| PcopriGM001959 | 4.7236 | 4.53E-05 | N-acetylmuramoyl-L-alanine amidase |
| PcopriGM003047 | 4.7236 | 4.53E-05 | hypothetical protein |
| PcopriGM000476 | 4.7187 | 6.38E-108 | glycoside hydrolase family 3 |
| PcopriGM002664 | 4.7086 | 2.33E-05 | hypothetical protein |
| PcopriGM000855 | 4.6878 | 6.05E-21 | outer membrane protein for nutrient uptake (SusD) |
| PcopriGM001823 | 4.6862 | 5.76E-05 | hypothetical protein |
| PcopriGM003067 | 4.6862 | 5.76E-05 | hypothetical protein |
| PcopriGM003251 | 4.6862 | 5.76E-05 | hypothetical protein |
| PcopriGM003118 | 4.6738 | 2.95E-05 | hypothetical protein |
| PcopriGM000948 | 4.665 | 8.27E-17 | hypothetical protein |
| PcopriGM000867 | 4.6477 | 7.31E-05 | endo-1 4-beta-xylanase |
| PcopriGM001134 | 4.6477 | 7.31E-05 | intron maturase |
| PcopriGM002119 | 4.6477 | 7.31E-05 | hypothetical protein |
| PcopriGM003216 | 4.6477 | 7.31E-05 | hypothetical protein |
| PcopriGM000256 | 4.6382 | 5.53E-09 | toxin-antitoxin system Bro |
| PcopriGM003233 | 4.6382 | 3.75E-05 | ATPase AAA |
| PcopriGM002102 | 4.6139 | 1.48E-12 | hypothetical protein |
| PcopriGM002287 | 4.6082 | 9.30E-05 | hypothetical protein |
| PcopriGM003284 | 4.6082 | 9.30E-05 | N-acetyltransferase |
| PcopriGM000859 | 4.6017 | 1.26E-30 | chromophore lyase |
| PcopriGM002095 | 4.5675 | 0.00011835 | hypothetical protein |
| PcopriGM002491 | 4.5675 | 0.00011835 | DUF3871 domain-containing protein |
| PcopriGM002808 | 4.5675 | 0.00011835 | hypothetical protein |
| PcopriGM000180 | 4.5257 | 0.00015078 | hypothetical protein |
| PcopriGM003280 | 4.5257 | 0.00015078 | hypothetical protein |
| PcopriGM001294 | 4.5159 | 3.33E-15 | TonB-dependent receptor |
| PcopriGM002974 | 4.5159 | 3.33E-15 | hypothetical protein |
| PcopriGM001977 | 4.4862 | 1.51E-11 | TonB-dependent receptor |
| PcopriGM000230 | 4.4831 | 1.05E-44 | hypothetical protein |
| PcopriGM002751 | 4.4826 | 0.00019227 | hypothetical protein |
| PcopriGM002755 | 4.4826 | 0.00019227 | hypothetical protein |
| PcopriGM000522 | 4.4455 | 5.80E-08 | hypothetical protein |
| PcopriGM000997 | 4.4455 | 0.0001251 | metallo-hydrolase |
| PcopriGM002048 | 4.4455 | 0.0001251 | hypothetical protein |
| PcopriGM000464 | 4.4382 | 0.00024542 | hypothetical protein |
| PcopriGM001688 | 4.4382 | 0.00024542 | hypothetical protein |
| PcopriGM002820 | 4.4382 | 0.00024542 | hypothetical protein |
| PcopriGM002093 | 4.4248 | 7.35E-08 | hypothetical protein |
| PcopriGM003125 | 4.4037 | 0.00015962 | DNA-binding protein |
| PcopriGM002993 | 4.3924 | 0.00031357 | hypothetical protein |
| PcopriGM001958 | 4.3824 | 1.18E-07 | hypothetical protein |
| PcopriGM002133 | 4.3824 | 6.88E-14 | recombinase |
| PcopriGM001442 | 4.3252 | 2.54E-16 | outer membrane protein for nutrient uptake (SusD) |
| PcopriGM000433 | 4.3163 | 0.00026059 | hypothetical protein |
| PcopriGM001295 | 4.3163 | 2.81E-13 | hypothetical protein |
| PcopriGM002111 | 4.3163 | 0.00026059 | glycosyl transferase |
| PcopriGM003124 | 4.3163 | 0.00026059 | DNA-binding protein |
| PcopriGM000383 | 4.2962 | 0.00051347 | hypothetical protein |
| PcopriGM002453 | 4.2962 | 0.00051347 | peptidase |
| PcopriGM003253 | 4.2962 | 0.00051347 | hypothetical protein |
| PcopriGM001110 | 4.247 | 4.95E-07 | redoxin domain protein |
| PcopriGM002371 | 4.247 | 3.06E-18 | outer membrane protein (SusF/SusE) |
| PcopriGM000224 | 4.2456 | 0.00065811 | hypothetical protein |
| PcopriGM001755 | 4.2456 | 0.00065811 | transcriptional repressor |
| PcopriGM002138 | 4.2456 | 0.00065811 | hypothetical protein |
| PcopriGM000047 | 4.2056 | 3.91E-31 | DUF2357 domain-containing protein |
| PcopriGM003149 | 4.1931 | 0.00084442 | ribonucleotide-diphosphate reductase |
| PcopriGM003283 | 4.1931 | 0.00084442 | hypothetical protein |
| PcopriGM001825 | 4.1742 | 0.00054743 | hypothetical protein |
| PcopriGM001999 | 4.1742 | 0.00054743 | hypothetical protein |
| PcopriGM001184 | 4.1576 | 2.72E-09 | glycosyl transferase |
| PcopriGM000263 | 4.1387 | 0.0010847 | hypothetical protein |
| PcopriGM000492 | 4.1387 | 0.0010847 | hypothetical protein |
| PcopriGM000477 | 4.1376 | 1.30E-29 | TonB-dependent receptor |
| PcopriGM002412 | 4.1236 | 1.65E-06 | mobilization protein |
| PcopriGM002988 | 4.1236 | 1.65E-06 | hypothetical protein |
| PcopriGM000650 | 4.0821 | 0.001395 | hypothetical protein |
| PcopriGM000847 | 4.0821 | 0.001395 | hypothetical protein |
| PcopriGM000942 | 4.0821 | 0.001395 | hypothetical protein |
| PcopriGM002397 | 4.0821 | 0.001395 | hybrid sensor histidine kinase |
| PcopriGM003166 | 4.0821 | 0.001395 | hypothetical protein |
| PcopriGM003277 | 4.0821 | 0.001395 | hypothetical protein |
| PcopriGM003282 | 4.0821 | 0.001395 | hypothetical protein |
| PcopriGM002809 | 4.0577 | 9.74E-21 | hypothetical protein |
| PcopriGM001895 | 4.0232 | 0.0017961 | n-acetylmuramoyl-L-alanine amidase |
| PcopriGM002097 | 4.0232 | 0.0017961 | hydrolase TatD |
| PcopriGM000896 | 4.0167 | 8.20E-11 | site-specific integrase |
| PcopriGM001781 | 4.0167 | 4.35E-06 | hypothetical protein |
| PcopriGM000848 | 3.9745 | 1.68E-10 | hypothetical protein |
| PcopriGM000401 | 3.9618 | 0.0023155 | hypothetical protein |
| PcopriGM002846 | 3.9618 | 0.0023155 | hypothetical protein |
| PcopriGM000915 | 3.9601 | 3.78E-08 | hypothetical protein |
| PcopriGM003324 | 3.9601 | 0.0014939 | hypothetical protein |
| PcopriGM002242 | 3.9012 | 0.0019253 | hypothetical protein |
| PcopriGM000045 | 3.8977 | 0.002989 | hypothetical protein |
| PcopriGM000443 | 3.8977 | 0.002989 | hypothetical protein |
| PcopriGM001390 | 3.8977 | 0.002989 | hypothetical protein |
| PcopriGM003340 | 3.8977 | 0.002989 | relaxase |
| PcopriGM002939 | 3.8708 | 4.50E-18 | TonB-dependent receptor |
| PcopriGM003235 | 3.8708 | 8.94E-10 | hypothetical protein |
| PcopriGM002991 | 3.8606 | 7.83E-14 | hypothetical protein |
| PcopriGM002090 | 3.8398 | 1.88E-05 | site-specific integrase |
| PcopriGM002364 | 3.8398 | 1.05E-29 | glycoside hydrolase, family 31 |
| PcopriGM003343 | 3.8398 | 0.0024842 | DUF3408 domain-containing protein |
| PcopriGM001051 | 3.8306 | 0.0038634 | flavodoxin |
| PcopriGM001678 | 3.8306 | 0.0038634 | nitrogen regulator |
| PcopriGM002099 | 3.8306 | 0.0038634 | hypothetical protein |
| PcopriGM002135 | 3.8306 | 0.0038634 | hypothetical protein |
| PcopriGM002143 | 3.8306 | 0.0038634 | hypothetical protein |
| PcopriGM003064 | 3.8306 | 0.0038634 | hypothetical protein |
| PcopriGM001441 | 3.8081 | 2.41E-05 | toxin-antitoxin system HipA |
| PcopriGM000050 | 3.8017 | 2.73E-11 | DNA methyltransferase |
| PcopriGM000339 | 3.792 | 4.81E-17 | glycoside hydrolase family 127 |
| PcopriGM002978 | 3.792 | 2.97E-09 | acetyl xylan esterase (AXE1) |
| PcopriGM000746 | 3.7757 | 3.32E-07 | hypothetical protein |
| PcopriGM001893 | 3.7757 | 0.0032091 | DNA-binding protein |
| PcopriGM002216 | 3.7757 | 0.0032091 | DUF3987 domain-containing protein |
| PcopriGM002460 | 3.7757 | 0.0032091 | hypothetical protein |
| PcopriGM000894 | 3.7602 | 0.0050004 | aminotransferase |
| PcopriGM003167 | 3.7602 | 0.0050004 | hypothetical protein |
| PcopriGM003170 | 3.7602 | 0.0050004 | hypothetical protein |
| PcopriGM000431 | 3.6987 | 4.25E-14 | galactopyranose mutase |
| PcopriGM000325 | 3.6862 | 0.0064812 | hypothetical protein |
| PcopriGM000838 | 3.6862 | 0.0064812 | hypothetical protein |
| PcopriGM002134 | 3.6862 | 0.0064812 | hypothetical protein |
| PcopriGM002958 | 3.6862 | 0.0064812 | hypothetical protein |
| PcopriGM003285 | 3.6862 | 0.0064812 | tetracycline resistance ribosomal protection protein |
| PcopriGM000863 | 3.662 | 1.30E-27 | glycoside hydrolase family 3 |
| PcopriGM001924 | 3.6082 | 0.008413 | hypothetical protein |
| PcopriGM002122 | 3.6082 | 0.008413 | hypothetical protein |
| PcopriGM002400 | 3.5893 | 2.32E-06 | beta-glucanase |
| PcopriGM000572 | 3.5642 | 0.0069697 | hypothetical protein |
| PcopriGM001761 | 3.5642 | 0.0069697 | hypothetical protein |
| PcopriGM000421 | 3.5347 | 7.40E-22 | hypothetical protein |
| PcopriGM000406 | 3.5335 | 3.69E-17 | transcriptional regulator LacI |
| PcopriGM000526 | 3.5257 | 0.00017392 | hypothetical protein |
| PcopriGM000570 | 3.5257 | 0.010937 | hypothetical protein |
| PcopriGM000851 | 3.5257 | 0.010937 | acetyl xylan esterase |
| PcopriGM001070 | 3.5257 | 0.010937 | hypothetical protein |
| PcopriGM001996 | 3.5257 | 0.00017392 | hypothetical protein |
| PcopriGM002187 | 3.5257 | 0.010937 | hypothetical protein |
| PcopriGM002494 | 3.5257 | 0.010937 | hypothetical protein |
| PcopriGM003171 | 3.5257 | 0.010937 | hypothetical protein |
| PcopriGM002100 | 3.5201 | 2.44E-12 | hypothetical protein |
| PcopriGM000858 | 3.4862 | 0.0090497 | hypothetical protein |
| PcopriGM001379 | 3.4862 | 0.00022307 | hypothetical protein |
| PcopriGM001644 | 3.4862 | 0.0090497 | ABC transporter |
| PcopriGM002554 | 3.4862 | 6.16E-06 | hypothetical protein |
| PcopriGM002184 | 3.4701 | 6.78E-09 | hypothetical protein |
| PcopriGM000368 | 3.4592 | 3.96E-19 | hypothetical protein |
| PcopriGM001181 | 3.4317 | 4.22E-10 | hypothetical protein |
| PcopriGM000893 | 3.4214 | 3.24E-62 | fructokinase |
| PcopriGM002372 | 3.4037 | 0.011767 | hypothetical protein |
| PcopriGM000886 | 3.3752 | 1.10E-09 | levanase |
| PcopriGM003208 | 3.3607 | 0.00047167 | hypothetical protein |
| PcopriGM001930 | 3.3442 | 9.40E-23 | peptidase ABC transporter |
| PcopriGM001156 | 3.3418 | 8.73E-11 | helicase |
| PcopriGM002089 | 3.3418 | 8.73E-11 | mobilization protein |
| PcopriGM002217 | 3.2859 | 3.42E-05 | hypothetical protein |
| PcopriGM001785 | 3.247 | 2.91E-11 | hypothetical protein |
| PcopriGM002088 | 3.247 | 2.56E-06 | hypothetical protein |
| PcopriGM003189 | 3.2232 | 5.58E-05 | hypothetical protein |
| PcopriGM001909 | 3.207 | 1.53E-08 | hypothetical protein |
| PcopriGM002685 | 3.2038 | 2.50E-07 | hypothetical protein |
| PcopriGM002990 | 3.1907 | 7.13E-05 | hypothetical protein |
| PcopriGM000500 | 3.1842 | 3.18E-07 | hypothetical protein |
| PcopriGM000004 | 3.1742 | 3.14E-15 | lipoprotein |
| PcopriGM001182 | 3.1643 | 4.04E-07 | transferase |
| PcopriGM001783 | 3.1528 | 2.46E-09 | glycosyl transferase |
| PcopriGM000511 | 3.1236 | 3.96E-09 | hypothetical protein |
| PcopriGM002101 | 3.0711 | 1.40E-05 | transposase |
| PcopriGM000048 | 3.046 | 9.11E-17 | hypothetical protein |
| PcopriGM002469 | 3.0167 | 2.08E-09 | DUF1738 domain-containing protein |
| PcopriGM002992 | 3.0167 | 2.27E-05 | hypothetical protein |
| PcopriGM003240 | 3.0167 | 0.0027379 | hypothetical protein |
| PcopriGM002687 | 2.9981 | 2.69E-07 | hypothetical protein |
| PcopriGM002853 | 2.9943 | 2.76E-06 | Transposase |
| PcopriGM000419 | 2.9887 | 8.67E-21 | outer membrane protein (SusC) |
| PcopriGM002370 | 2.9816 | 7.67E-17 | glycoside hydrolase, family 66 |
| PcopriGM003293 | 2.9665 | 5.35E-10 | sugar phosphate isomerase |
| PcopriGM000087 | 2.9601 | 8.84E-13 | pectinesterase |
| PcopriGM002425 | 2.9601 | 5.35E-09 | conjugative transposon protein TraM |
| PcopriGM001906 | 2.9557 | 1.14E-13 | pectate lyase |
| PcopriGM002985 | 2.9112 | 2.85E-12 | hypothetical protein |
| PcopriGM000945 | 2.9012 | 4.62E-13 | outer membrane protein (SusC) |
| PcopriGM001881 | 2.8792 | 4.48E-11 | hypothetical protein |
| PcopriGM000007 | 2.8692 | 6.91E-18 | TonB-dependent receptor |
| PcopriGM002688 | 2.8606 | 0.00065044 | hypothetical protein |
| PcopriGM001786 | 2.8488 | 2.23E-07 | hypothetical protein |
| PcopriGM000852 | 2.8398 | 3.53E-08 | acetyl xylan esterase |
| PcopriGM000937 | 2.8398 | 3.53E-08 | rhamnulose-1-phosphate aldolase |
| PcopriGM002464 | 2.8308 | 2.82E-07 | hypothetical protein |
| PcopriGM000999 | 2.8287 | 1.31E-15 | hypothetical protein |
| PcopriGM000006 | 2.8127 | 3.58E-07 | outer membrane protein for nutrient uptake (SusD) |
| PcopriGM001774 | 2.7974 | 5.37E-16 | hypothetical protein |
| PcopriGM000049 | 2.7757 | 9.02E-08 | DNA methyltransferase |
| PcopriGM001179 | 2.7757 | 9.02E-08 | polysaccharide pyruvyl transferase |
| PcopriGM001694 | 2.7757 | 0.0075243 | hypothetical protein |
| PcopriGM000653 | 2.7425 | 0.00019976 | hypothetical protein |
| PcopriGM000946 | 2.7313 | 5.89E-06 | outer membrane protein for nutrient uptake (SusD) |
| PcopriGM001631 | 2.7313 | 0.0013581 | hypothetical protein |
| PcopriGM000914 | 2.7223 | 1.80E-16 | outer membrane protein for nutrient uptake (SusD) |
| PcopriGM003342 | 2.7175 | 1.25E-18 | intron reverse transcriptase |
| PcopriGM001129 | 2.7086 | 0.0096923 | Ribonuclease |
| PcopriGM002624 | 2.6738 | 0.0003233 | hypothetical protein |
| PcopriGM000393 | 2.662 | 2.01E-18 | alpha-amylase |
| PcopriGM001766 | 2.6382 | 1.51E-05 | hypothetical protein |
| PcopriGM003292 | 2.6174 | 3.74E-06 | hypothetical protein |
| PcopriGM002806 | 2.6139 | 1.24E-17 | hypothetical protein |
| PcopriGM001787 | 2.5942 | 1.09E-14 | lipopolysaccharide biosynthesis |
| PcopriGM000407 | 2.5915 | 5.78E-16 | MFS transporter |
| PcopriGM001775 | 2.5893 | 2.33E-07 | glycosyl transferase |
| PcopriGM003187 | 2.5387 | 0.0036158 | hypothetical protein |
| PcopriGM001956 | 2.5344 | 8.41E-32 | hypothetical protein |
| PcopriGM002997 | 2.5257 | 0.00084405 | DUF2149 domain-containing protein |
| PcopriGM001836 | 2.5039 | 7.37E-07 | Recombination protein O |
| PcopriGM000420 | 2.4683 | 1.17E-06 | outer membrane protein for nutrient uptake (SusD) |
| PcopriGM000839 | 2.4683 | 6.21E-12 | cellulase, GH5 |
| PcopriGM002733 | 2.4631 | 1.91E-05 | hypothetical protein |
| PcopriGM001760 | 2.4603 | 7.10E-16 | hypothetical protein |
| PcopriGM003181 | 2.4509 | 1.34E-14 | metallo-beta-lactamase |
| PcopriGM000862 | 2.4501 | 9.73E-12 | alpha-glucosidase, GH31 |
| PcopriGM000943 | 2.4006 | 4.37E-16 | TonB-dependent receptor |
| PcopriGM002625 | 2.3752 | 0.0075136 | N-acetylmuramoyl-L-alanine amidase |
| PcopriGM000654 | 2.346 | 1.14E-10 | hypothetical protein |
| PcopriGM000152 | 2.3433 | 2.84E-18 | beta-galactosidase, GH2 |
| PcopriGM000913 | 2.338 | 3.54E-18 | outer membrane protein (SusC) |
| PcopriGM002964 | 2.3362 | 3.99E-15 | hypothetical protein |
| PcopriGM003330 | 2.3342 | 1.79E-06 | intron reverse transcriptase |
| PcopriGM000938 | 2.3163 | 7.22E-06 | rhamnose proton symporter RhaT |
| PcopriGM002290 | 2.3163 | 2.19E-09 | hypothetical protein |
| PcopriGM002516 | 2.3163 | 0.00082433 | hypothetical protein |
| PcopriGM002540 | 2.3163 | 0.00082433 | hypothetical protein |
| PcopriGM003321 | 2.3163 | 2.74E-18 | hypothetical protein |
| PcopriGM001926 | 2.3012 | 2.76E-07 | hypothetical protein |
