# Supplementary material for: Isolation of Low-Abundant Bacteroidales in the Human Intestine and the Analysis of Their Differential Utilization Based on Plant-Derived Polysaccharides
Source: Front Microbiol. 2018 Jun 19;9:1319. doi: 10.3389/fmicb.2018.01319 (PMC6018473; doi:10.3389/fmicb.2018.01319)
Supplement: Supplementary file 2 [file Table_2.DOCX]

Table S2 Genes repressed over 5-fold in *P. copri* ELH-XY3 during fermentation in xylan relative to xylose. Genes are listed by magnitude of induction. Gene annotation was carried out by blastp against the NCBI database.

| Gene_id | Fold Change (log2) | p-value | annotation |
| --- | --- | --- | --- |
| PcopriGM000454 | 5.6837 | 4.20E-30 | sensor histidine kinase KdpD |
| PcopriGM003108 | 4.7997 | 2.11E-203 | beta-galactosidase |
| PcopriGM001488 | 3.3056 | 1.25E-22 | hypothetical protein |
| PcopriGM000607 | 3.0139 | 1.67E-13 | hypothetical protein |
| PcopriGM003046 | 2.7426 | 0.0091652 | type IV secretion protein Rhs |
| PcopriGM000903 | 2.7094 | 5.94E-134 | hypothetical protein |
| PcopriGM000080 | 2.5417 | 0.0079841 | hypothetical protein |
| PcopriGM001223 | 2.5009 | 4.22E-13 | sugar transferase |
| PcopriGM002056 | 2.4141 | 1.09E-08 | hypothetical protein |
| PcopriGM000305 | 2.4034 | 2.30E-16 | hypothetical protein |
| PcopriGM000202 | 2.3199 | 9.17E-19 | Biopolymer transport protein |
