# Supplementary material for: Isolation of Low-Abundant Bacteroidales in the Human Intestine and the Analysis of Their Differential Utilization Based on Plant-Derived Polysaccharides
Source: Front Microbiol. 2018 Jun 19;9:1319. doi: 10.3389/fmicb.2018.01319 (PMC6018473; doi:10.3389/fmicb.2018.01319)
Supplement: Supplementary file 3 [file Table_3.DOCX]

Table S3 Genes induced over 5-fold in *B. xylanisolvens* HCM-XY17 during fermentation in xylan relative to xylose. Genes are listed by magnitude of induction. Gene annotation was carried out by blastp against the NCBI database.

| Gene_id | Fold Change (log2) | p-value | annotation |
| --- | --- | --- | --- |
| BxylanGM003581 | 6.8389 | 5.14E-14 | outer membrane protein for nutrient uptake (SusD) |
| BxylanGM003881 | 4.8558 | 2.93E-23 | peptide transporter |
| BxylanGM000909 | 4.8396 | 3.84E-27 | reductase |
| BxylanGM003304 | 4.8195 | 3.89E-17 | outer membrane protein (SusC) |
| BxylanGM003305 | 4.7535 | 5.68E-14 | outer membrane protein for nutrient uptake (SusD) |
| BxylanGM000912 | 4.6842 | 1.32E-09 | hypothetical protein |
| BxylanGM003303 | 4.5601 | 3.85E-14 | ribokinase |
| BxylanGM000911 | 4.5379 | 8.73E-22 | L-fucose:H+ symporter permease |
| BxylanGM000240 | 4.3978 | 0.00012229 | RNA polymerase |
| BxylanGM003302 | 4.388 | 1.21E-11 | ribokinase |
| BxylanGM003309 | 4.2927 | 1.33E-29 | MFS transporter |
| BxylanGM002171 | 4.2615 | 1.67E-07 | outer membrane protein (SusE) |
| BxylanGM003308 | 4.1095 | 9.45E-22 | EbgC protein |
| BxylanGM003310 | 4.0788 | 2.13E-30 | glycoside hydrolase, family 32 |
| BxylanGM002174 | 4.0781 | 2.12E-07 | glycoside hydrolase, family 92 |
| BxylanGM002170 | 3.9246 | 8.89E-06 | glycoside hydrolase, family 76 |
| BxylanGM003306 | 3.9197 | 8.62E-18 | dihydrodipicolinate synthase |
| BxylanGM000910 | 3.9104 | 4.90E-15 | amidohydrolase |
| BxylanGM003307 | 3.884 | 1.52E-25 | dihydrodipicolinate synthase |
| BxylanGM002169 | 3.8082 | 2.18E-07 | TonB-dependent receptor |
| BxylanGM002168 | 3.7452 | 2.44E-08 | outer membrane protein for nutrient uptake (SusD) |
| BxylanGM002167 | 3.6026 | 1.40E-07 | hypothetical protein |
| BxylanGM003003 | 3.3342 | 2.02E-26 | urocanate hydratase |
| BxylanGM003877 | 3.0311 | 3.16E-07 | hypothetical protein |
| BxylanGM001449 | 3.0254 | 1.20E-05 | hypothetical protein |
| BxylanGM000282 | 3.0147 | 5.84E-10 | hydrogenases |
| BxylanGM003274 | 3.0026 | 0.00012198 | ATPase |
| BxylanGM003272 | 2.9581 | 0.00021628 | hypothetical protein |
| BxylanGM002172 | 2.9355 | 3.90E-06 | hypothetical protein |
| BxylanGM001549 | 2.9345 | 1.78E-21 | pyrophosphate-energized sodium pump |
| BxylanGM000895 | 2.7772 | 1.12E-11 | outer membrane protein for nutrient uptake (SusD) |
| BxylanGM001065 | 2.7335 | 0.0030584 | sugar phosphate isomerase |
| BxylanGM003880 | 2.697 | 2.62E-08 | hypothetical protein |
| BxylanGM004413 | 2.6948 | 0.00012809 | hypothetical protein |
| BxylanGM003097 | 2.6628 | 2.21E-10 | DUF3408 domain-containing protein |
| BxylanGM002349 | 2.6231 | 4.37E-16 | outer membrane protein (SusC) |
| BxylanGM000898 | 2.6166 | 3.67E-09 | hypothetical protein |
| BxylanGM002968 | 2.6061 | 1.37E-05 | outer membrane protein for nutrient uptake (SusD) |
| BxylanGM002350 | 2.5564 | 4.70E-07 | outer membrane protein for nutrient uptake (SusD) |
| BxylanGM003111 | 2.5495 | 2.07E-13 | conjugal transfer protein |
| BxylanGM003105 | 2.5441 | 2.35E-13 | DUF4141 domain-containing protein |
| BxylanGM000896 | 2.5324 | 1.90E-10 | TonB-dependent receptor |
| BxylanGM002967 | 2.5137 | 1.28E-05 | glucosylceramidase |
| BxylanGM000283 | 2.5122 | 3.32E-05 | hydrogenases |
| BxylanGM002966 | 2.4944 | 3.04E-05 | DUF5125 domain-containing protein |
| BxylanGM004415 | 2.482 | 8.01E-13 | DUF4099 domain-containing protein |
| BxylanGM000893 | 2.4382 | 1.58E-07 | beta-hexosaminidase |
| BxylanGM004493 | 2.4379 | 1.43E-05 | outer membrane protein (SusC) |
| BxylanGM003107 | 2.4293 | 7.07E-14 | conjugative transposon protein |
| BxylanGM003290 | 2.4235 | 5.14E-10 | outer membrane protein for nutrient uptake (SusD) |
| BxylanGM002377 | 2.4228 | 7.91E-06 | hypothetical protein |
| BxylanGM002975 | 2.4079 | 0.0053231 | hypothetical protein |
| BxylanGM003092 | 2.3945 | 5.88E-06 | mobilization protein |
| BxylanGM003275 | 2.3675 | 0.00051738 | ATPase |
| BxylanGM004402 | 2.3662 | 2.15E-06 | DNA-binding protein |
| BxylanGM002198 | 2.3612 | 0.00027992 | hypothetical protein |
| BxylanGM004401 | 2.3565 | 5.40E-08 | DNA-binding protein |
| BxylanGM004492 | 2.3528 | 0.0017995 | outer membrane protein for nutrient uptake (SusD) |
| BxylanGM000778 | 2.3503 | 8.10E-13 | ABC transporter permease |
| BxylanGM003580 | 2.3469 | 2.49E-15 | alpha-1,2-mannosidase |
| BxylanGM003095 | 2.3426 | 3.22E-05 | hypothetical protein |
| BxylanGM001974 | 2.3405 | 0.0040148 | DUF4248 domain-containing protein |
| BxylanGM003112 | 2.3277 | 4.15E-08 | DUF3872 domain-containing protein |
| BxylanGM003113 | 2.3193 | 7.93E-12 | glycoside hydrolase |
