# Supplementary material for: Isolation of Low-Abundant Bacteroidales in the Human Intestine and the Analysis of Their Differential Utilization Based on Plant-Derived Polysaccharides
Source: Front Microbiol. 2018 Jun 19;9:1319. doi: 10.3389/fmicb.2018.01319 (PMC6018473; doi:10.3389/fmicb.2018.01319)
Supplement: Supplementary file 4 [file Table_4.DOCX]

Table S4 Genes repressed over 5-fold in *B. xylanisolvens* HCM-XY17 during fermentation in xylan relative to xylose. Genes are listed by magnitude of induction. Gene annotation was carried out by blastp against the NCBI database.

| Gene_id | Fold Change (log2) | p-value | annotation |
| --- | --- | --- | --- |
| BxylanGM003886 | 3.8595 | 1.79E-12 | bifunctional hydroxymethylpyrimidine kinase |
| BxylanGM000584 | 3.5705 | 5.43E-06 | hypothetical protein |
| BxylanGM002583 | 3.0523 | 0.003512 | threonine/serine exporter |
| BxylanGM002584 | 2.7021 | 0.00085199 | threonine/serine exporter |
| BxylanGM000285 | 2.5746 | 0.00078041 | acetyltransferase |
| BxylanGM000286 | 2.4937 | 0.00039445 | pyruvate formate lyase |
| BxylanGM002580 | 2.3002 | 9.64E-05 | phosphatase |
