# Supplementary material for: Isolation of Low-Abundant Bacteroidales in the Human Intestine and the Analysis of Their Differential Utilization Based on Plant-Derived Polysaccharides
Source: Front Microbiol. 2018 Jun 19;9:1319. doi: 10.3389/fmicb.2018.01319 (PMC6018473; doi:10.3389/fmicb.2018.01319)
Supplement: Supplementary file 5 [file Table_5.DOCX]

Table S5 Genes induced over 5-fold in *B. uniformis* HCM-XY15 during fermentation in xylan relative to xylose. Genes are listed by magnitude of induction. Gene annotation was carried out by blastp against the NCBI database.

| Gene_id | Fold Change (log2) | p-value | annotation |
| --- | --- | --- | --- |
| BuniforGM002564 | 7.3884 | 0.00032463 | 1,4-alpha-glucan branching enzyme |
| BuniforGM002567 | 5.6579 | 0.00074463 | 1,4-alpha-glucan branching enzyme |
| BuniforGM002568 | 5.2636 | 0.001814 | arabinogalactan endo-1,4-beta-galactosidase, GH53 |
| BuniforGM002052 | 4.8279 | 1.03E-26 | hypothetical protein |
| BuniforGM002410 | 4.6558 | 0.0020814 | 1,4-alpha-glucan branching enzyme |
| BuniforGM000762 | 4.1099 | 0.0015634 | hypothetical protein |
| BuniforGM002050 | 3.9485 | 3.84E-13 | hypothetical protein |
| BuniforGM002842 | 3.8919 | 0.0036575 | Efflux pump membrane transporter |
| BuniforGM002046 | 3.7479 | 2.40E-07 | hypothetical protein |
| BuniforGM002400 | 3.7479 | 0.00016517 | aspartate 4-decarboxylase |
| BuniforGM002843 | 3.7316 | 0.0022805 | efflux RND transporter periplasmic adaptor |
| BuniforGM002051 | 3.5193 | 6.27E-16 | hypothetical protein |
| BuniforGM003186 | 3.4057 | 0.0020184 | ABC transporter permease |
| BuniforGM002068 | 3.3961 | 6.24E-20 | hypothetical protein |
| BuniforGM003185 | 3.322 | 0.0029321 | hemolysin secretion protein |
| BuniforGM002070 | 3.2974 | 1.60E-07 | terminase |
| BuniforGM002065 | 3.2637 | 5.66E-08 | hypothetical protein |
| BuniforGM002042 | 3.2338 | 2.98E-06 | hypothetical protein |
| BuniforGM002585 | 3.2243 | 6.12E-05 | TolC family protein |
| BuniforGM002063 | 3.2172 | 2.61E-13 | hypothetical protein |
| BuniforGM003184 | 3.1798 | 0.00084846 | TolC family protein |
| BuniforGM002061 | 3.172 | 6.11E-15 | hypothetical protein |
| BuniforGM002584 | 3.1284 | 0.00022985 | AcrB/AcrD/AcrF family protein |
| BuniforGM002399 | 3.0808 | 0.00048726 | aspartate-alanine antiporter |
| BuniforGM000496 | 3.0275 | 6.79E-07 | hypothetical protein |
| BuniforGM002058 | 3.0248 | 2.57E-10 | hypothetical protein |
| BuniforGM002047 | 3.0202 | 4.69E-11 | hypothetical protein |
| BuniforGM002067 | 2.9787 | 1.37E-07 | hypothetical protein |
| BuniforGM003902 | 2.9591 | 1.70E-06 | hypothetical protein |
| BuniforGM002064 | 2.8901 | 7.13E-05 | hypothetical protein |
| BuniforGM002066 | 2.8747 | 1.33E-16 | hypothetical protein |
| BuniforGM001165 | 2.8643 | 6.20E-05 | outer membrane protein (SusC) |
| BuniforGM003899 | 2.8351 | 7.13E-08 | hypothetical protein |
| BuniforGM002060 | 2.8166 | 0.0007292 | hypothetical protein |
| BuniforGM002583 | 2.8121 | 1.28E-05 | efflux RND transporter periplasmic adaptor |
| BuniforGM001164 | 2.7346 | 0.0044726 | outer membrane protein for nutrient uptake (SusD) |
| BuniforGM002471 | 2.7197 | 0.00032198 | DUF4251 domain-containing protein |
| BuniforGM002071 | 2.6738 | 4.95E-06 | phage portal protein |
| BuniforGM002048 | 2.6449 | 2.46E-06 | hypothetical protein |
| BuniforGM000782 | 2.6406 | 0.0046167 | hydroxylamine reductase |
| BuniforGM002062 | 2.6323 | 7.38E-08 | hypothetical protein |
| BuniforGM002623 | 2.607 | 0.0033889 | outer membrane protein (SusC) |
| BuniforGM000908 | 2.5111 | 0.00010741 | DUF3575 domain-containing protein |
| BuniforGM002037 | 2.5065 | 0.00011593 | hypothetical protein |
| BuniforGM003466 | 2.4472 | 9.45E-07 | hypothetical protein |
| BuniforGM003725 | 2.4271 | 0.00035916 | MFS transporter |
| BuniforGM003472 | 2.4206 | 0.00012589 | hypothetical protein |
| BuniforGM000064 | 2.3838 | 3.31E-09 | outer membrane protein |
| BuniforGM002078 | 2.3636 | 2.41E-10 | primase |
| BuniforGM002055 | 2.3469 | 6.45E-07 | hypothetical protein |
| BuniforGM003479 | 2.3468 | 3.36E-08 | hypothetical protein |
| BuniforGM000633 | 2.3412 | 3.18E-05 | hypothetical protein |
