# Supplementary material for: Isolation of Low-Abundant Bacteroidales in the Human Intestine and the Analysis of Their Differential Utilization Based on Plant-Derived Polysaccharides
Source: Front Microbiol. 2018 Jun 19;9:1319. doi: 10.3389/fmicb.2018.01319 (PMC6018473; doi:10.3389/fmicb.2018.01319)
Supplement: Supplementary file 6 [file Table_6.DOCX]

Table S6 Genes repressed over 5-fold in *B. uniformis* HCM-XY15 during fermentation in xylan relative to xylose. Genes are listed by magnitude of induction. Gene annotation was carried out by blastp against the NCBI database.

| Gene_id | Fold Change (log2) | p-value | annotation |
| --- | --- | --- | --- |
| BuniforGM001189 | 5.0503 | 8.95E-41 | transketolase |
| BuniforGM001743 | 4.6672 | 1.86E-38 | glycosyl hydrolase |
| BuniforGM002577 | 3.8991 | 2.49E-05 | outer membrane protein for nutrient uptake (SusD) |
| BuniforGM001148 | 3.5295 | 7.29E-13 | outer membrane protein for nutrient uptake (SusD) |
| BuniforGM001147 | 3.3978 | 3.47E-17 | outer membrane protein (SusC) |
| BuniforGM002579 | 2.9847 | 3.04E-18 | hybrid sensor histidine kinase |
| BuniforGM000986 | 2.9221 | 6.76E-17 | outer membrane protein (SusC) |
| BuniforGM001460 | 2.8393 | 2.05E-06 | hypothetical protein |
| BuniforGM001048 | 2.8275 | 1.73E-12 | hypothetical protein |
| BuniforGM002578 | 2.7726 | 0.00026106 | outer membrane protein (SusC) |
| BuniforGM000606 | 2.6946 | 0.0012088 | hypothetical protein |
| BuniforGM003540 | 2.6883 | 3.99E-15 | alpha-N-arabinofuranosidase |
| BuniforGM000205 | 2.5209 | 4.29E-11 | alpha-L-arabinofuranosidase |
| BuniforGM003546 | 2.4136 | 6.85E-13 | galactose mutarotase |
| BuniforGM001149 | 2.4116 | 3.61E-10 | glycosyl hydrolase |
| BuniforGM000987 | 2.3772 | 4.27E-13 | outer membrane protein for nutrient uptake (SusD) |
| BuniforGM003545 | 2.376 | 5.53E-13 | hypothetical protein |
| BuniforGM001890 | 2.3539 | 2.19E-13 | xylose isomerase |
| BuniforGM001697 | 2.339 | 3.45E-10 | outer membrane protein for nutrient uptake (SusD) |
| BuniforGM001889 | 2.3318 | 3.64E-13 | DUF1080 domain-containing protein |
| BuniforGM000653 | 2.3223 | 0.0016962 | ABC transporter |
| BuniforGM001888 | 2.3127 | 3.14E-13 | hypothetical protein |
